# Identification and Validation of *RELN* Mutation as a Response Indicator for Immune Checkpoint Inhibitor Therapy in Melanoma and Non-Small Cell Lung Cancer

**DOI:** 10.3390/cells11233841

**Published:** 2022-11-30

**Authors:** Zhenpeng Li, Xin Wang, Yi Yang, Fuyan Shi, Wenjing Zhang, Qinghua Wang, Suzhen Wang

**Affiliations:** 1 School of Medical Laboratory, Weifang Medical University, Weifang 261053, China; 2 The Second Affiliated Hospital of Chongqing Medical University, Chongqing 400000, China; 3 Department of Health Statistics, Key Laboratory of Medicine and Health of Shandong Province, School of Public Health, Weifang Medical University, Weifang 261053, China

**Keywords:** *RELN* mutations, immune checkpoint inhibitor, melanoma, non-small cell lung cancer, molecular determinant

## Abstract

Remarkable clinical benefits in several advanced cancers are observed under the treatment of immune checkpoint inhibitor (ICI) agents. However, only a smaller proportion of patients respond to the treatments. Reelin (RELN) is frequently mutated in the cancer genome. In this study, the *RELN* mutation association with ICI treatment efficacy in melanoma and non-small cell lung cancer (NSCLC) was elucidated. Data from 631 melanoma and 109 NSCLC patients with both ICI treatment data and pre-treatment mutational profiles were collected. In addition, from the Cancer Genome Atlas (TCGA) project, we also obtained both tumors to explore the immunologic features behind *RELN* mutations. Melanoma patients with *RELN* mutations exhibited a favorable ICI survival benefit when compared with wild-type patients (HR: 0.66, 95% CI: 0.51–0.87, *p* = 0.003). A higher response rate was also noticed in *RELN*-mutated patients (38.9% vs. 28.3%, *p* = 0.017). The association of *RELN* mutations with a preferable immunotherapy outcome and response was further confirmed in NSCLC. Further exploration demonstrated that favorable immunocyte infiltration and immune response signaling pathways were found in patients with *RELN* mutations. In this study, *RELN* mutations were identified to connect with a better immune microenvironment and an improved ICI efficacy in melanoma and NSCLC, which provides a potential biomarker for immunological feature evaluation and immunotherapeutic outcome prediction at the molecular level.

## 1. Introduction

Recently, mutations in a single gene were reported to act as potential indicators for tumor immunogenicity and immunotherapeutic response. Li et al. leveraged the somatic mutational profiles of gastric cancer (GC) patients and found that *MUC16* mutations were linked with an elevated TMB and immunogenicity-related signaling pathways [1]. Further analysis showed that *MUC16*-mutated GC patients exhibited an improved survival outcome, which confirms the clinical immunotherapy significance of *MUC16* mutations [1]. Consistent with Li et al.’s results, two recent studies [2,3] validated that *MUC16* mutations were predictive of a favorable ICI treatment response and outcome. Mutations in *POLE* [4], *FAT1* [5], *TP53* [6], *COL3A1* [7], and *HSPG2* [8] were also identified to be associated with a better ICI prognosis or response rate. In addition, ICI treatment resistance was observed in patients with mutations of *JAK1*/*2* [9,10] or *B2M* [11].

Immune checkpoint inhibitors (ICIs) directing the programmed cell death (ligand) 1 [PD-(L)1] and cytotoxic T lymphocyte antigen 4 (CTLA-4) signals have become the routine clinical treatment regimens for multiple cancer types [12,13]. However, durable clinical response to ICI treatments has been observed only in a few patients [14]. Therefore, multiple biomarkers are determined to assist in selecting patients who will respond effectively to ICI treatments. Several biomarkers have been identified for predicting immunotherapeutic efficacy, such as tumor mutation burden (TMB) [15], PD-L1 protein expression on tumor cells [16], and neoantigen burden [17,18]. Nevertheless, many shortcomings have emerged regarding the above biomarkers in clinical practice; for instance, the uncertain cutoff value, the cost of genomic sequencing is too high, and the predictive rate is unsatisfactory, limiting the wide utilization of these markers in cancer immunotherapy [19]. Therefore, more effective immunotherapeutic determinants are urgently necessary for guiding treatment efficacy.

Reelin (RELN) encodes a large secreted extracellular matrix protein, and it is critical for cell positioning. Studies conducted in the null reeler mouse have indicated that the serine protease activity of RELN is critical for developing the brain [19]. Pathways triggered by RELN depend on the recruitment of distinct cell surface receptors, i.e., very low density lipoprotein receptor (VLDLR) [20], apolipoprotein E receptor 2 (ApoER2 or LRP8) [20,21], α3β1 integrin [22], and members of the cadherin-related neuronal receptor (CNR) family [23]. A number of studies have revealed changes in the expression of RELN in different cancer types [24]. RELN expression has been observed to reduce in breast [25], colorectal [26], and pancreatic cancers [27], while it has been noticed to be increased in retinoblastoma [28], myelomas [29], and prostate cancers [30]. To our knowledge, the immunological and cancer immune treatment implications of *RELN* mutations have not been reported in clinical practice.

Since melanoma and NSCLC are two cancer types commonly used immunotherapies, in this genomic association analysis, we retrospectively integrated a total of 631 melanoma and 119 NSCLC samples; their pre-treatment mutational data and clinical ICI therapy information were also obtained. We discovered that *RELN* mutations may be a potential biomarker for cancer immunotherapeutic efficacy prediction.

## 2. Materials and Methods

### 2.1. Sample Collection and Genomic Data Processing

From previously published melanoma [10,31,32,33,34,35,36,37] and NSCLC [38,39] immunogenomic studies, we retrospectively collected a total of 631 and 109 samples, with both somatic mutational data and ICI treatment information (i.e., response and survival information). All the above samples were treated with checkpoint blockade treatments of CTLA-4, PD-1/PD-L1, or a combination drug. Taking into account that the mutational data were obtained from distinct sequencing platforms, we therefore uniformly unscrambled them with the Oncotator [40]. In this work, non-synonymous mutations were included for the subsequent analyses. Detailed clinical data and immunotherapy response information for curated melanoma and NSCLC samples are exhibited in Tables S1 and S2, respectively.

A total of 457 melanoma and 995 NSCLC cases with mutational data, gene expression profiles, and clinicopathological information in the Cancer Genome Atlas (TCGA) cohort were acquired from Genome Data Commons (https://gdc.cancer.gov, accessed on 1 September 2022). In particular, the gene expression profiles of both tumors in the TCGA were employed for the immunological mechanism exploration of *RELN* mutations.

### 2.2. Detection of Tumor Mutational Signatures

Tumor mutational signatures were determined by using a nonnegative matrix factorization-based algorithm proposed by a recent study [41], which could divide the somatic mutational matrix *A* into 2 nonnegative matrices *W* and *H* (i.e., *A* ≈ *W* × *H*). Of these, *W* indicates the determined mutational signatures and *H* indicates the mutational activities for each signature. All identified signatures were then compared with 30 well-annotated signatures reserved in the COSMIC (version 2, https://cancer.sanger.ac.uk/cosmic, accessed on 1 September 2022) based on the cosine similarity.

### 2.3. Infiltration Abundance of Immune Cell Subtypes

We used the CIBERSORT algorithm [42] to conclude the distinct infiltration levels of 22 immunocytes in *RELN*-mutant and wild-type subgroups. A total of 547 feature genes for the above immune cells, termed LM22 signature within the CIBERSORT, were employed to evaluate infiltration levels.

### 2.4. IFNγ-Related Gene Signature

Interferon γ (IFNγ) signature [43] includes immune genes (i.e., *GBP1*, *IFI16*, *IFI30*, *IFNG*, *IRF1*, *STAT1*, *TAP1*, *TAP2*, *PSMB9*, *IL15RA*, *GZMA*, *GZMB*, *CXCL10, CXCL9*, and *TBX21*) associated with antigen presentation, cytotoxic activity, and adaptive immune response. A previous study has demonstrated that this T cell-inflamed gene expression signature could serve as an indicator for quantifying tumor microenvironment and is predictive of the clinical response to anti-PD-1 therapies.

### 2.5. GSVA and GSEA

We performed differential expression analysis of the whole genome between *RELN*-mutated and wild-type subgroups by using the DESeq2 R package [44]. All genes with their corresponding *t* values obtained from differential analysis were put into *fgsea* functions embedded in R fgsea packages to conduct gene set enrichment analysis (GSEA). Signaling pathways in the Hallmark database were utilized to infer dysregulated pathways. In addition, in order to calculate the enrichment scores of IFNγ signature for each sample with specific feature genes, a single sample GSEA method in R GSVA package [45] was used.

### 2.6. Statistical Analysis

R software (version 4.2.1) was used in this study to complete related analyses and plots. Mutational patterns for specific genes were illustrated with a waterfall plot under R maftools package [46]. In this analysis, TMB was defined as the log2 transformation of total non-synonymous mutations per megabase in both tumors. The Kaplan-Meier method was used to achieve survival curves and the log-rank test was used to compare the survival difference significance. Multivariable regression analyses (i.e., logistic and Cox regression) with multiple confounding factors taken into account were performed with R forestmodel package. *RELN* mutation associations with continuous and categorical variables were evaluated with Wilcoxon rank-sum test and Fisher exact test, respectively.

## 3. Results

### 3.1. ICI Response Information for Melanoma Patients and RELN Mutations

**Figure 1:**
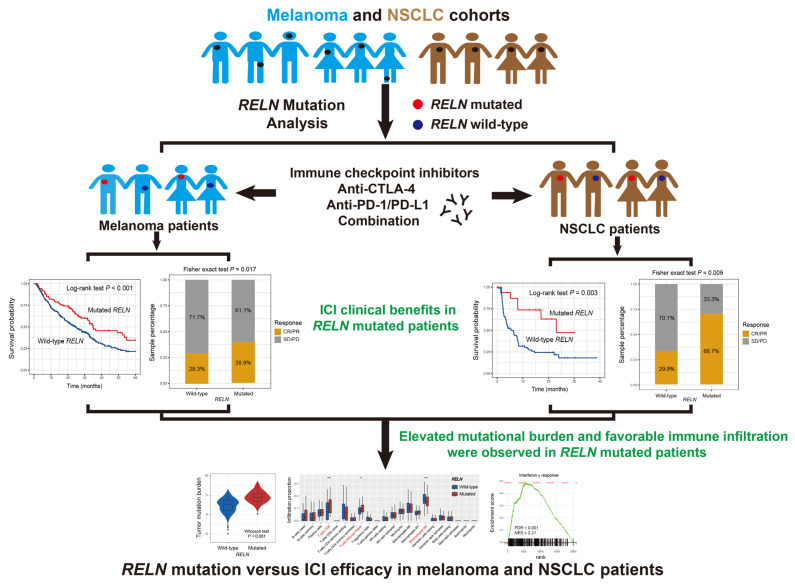
The detailed workflow operating in this work to explore the clinical ICI treatment implications of *RELN* mutations based on the genomic data and immunotherapy information.

The detailed workflow of this study is shown in Figure 1. A total of 631 melanoma patients were included in this study, of which 193 (30.6%) exhibited the ICI response statuses (i.e., complete response or partial response), 430 (68.1%) were non-responders (i.e., stable disease or progressive disease), and the rest (1.3%) were unavailable. The mutational waterfall plot showed that C > T mutations were the primary base substitution pattern in the melanoma cohort (Figure S1). Mutational patterns of *RELN* and frequently mutated driver genes in melanoma are illustrated in Figure S1. A total of 160 of the 631 patients (25.4%) harbored *RELN* mutations and *RELN* mutation-induced amino acid changes are exhibited using a lollipop plot in Figure S2.

### 3.2. RELN Mutations in Predicting ICI Treatment Efficacy in Melanoma

**Figure 2:**
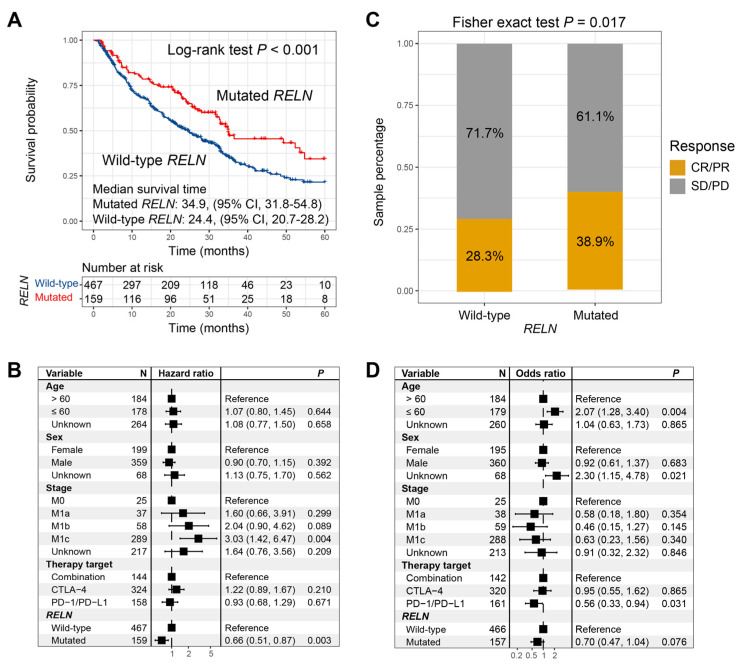
ICI treatment prognosis and response rate analyses of *RELN* mutations in melanoma. (**A**) Survival curves of *RELN*-mutated and wild-type patients. (**B**) Multivariable Cox regression analysis of *RELN* mutations was performed with clinical confounders taken into consideration. (**C**) Bar plot representation of ICI response rates of *RELN*-mutated and wild-type patients. (**D**) Multivariable logistic regression analysis of *RELN* mutations was achieved.

Significant ICI survival benefits were observed in melanoma patients who harbored *RELN* mutations (median survival time: 34.9 vs. 24.4 months, Log-rank test *p* < 0.001; Figure 2A). We further incorporated multiple confounding factors (e.g., age, sex, stage, and therapy type) into a multivariable Cox regression analysis, and the association between *RELN* mutations and favorable ICI survival was still noticed (HR: 0.66, 95% CI: 0.51–0.87, *p* = 0.003; Figure 2B). Roles of *RELN* mutations in evaluating ICI treatment prognosis in included single cohorts, and distinct treatment types are illustrated in Figures S3 and S4. Further analysis demonstrated that *RELN* mutations were also connected with a significantly elevated ICI response rate (38.9% vs. 28.3%, Fisher exact test *p* = 0.017; Figure 2C). a multivariable logistic regression analysis with the confounding variables taken into account still revealed a positive association (OR: 0.70, 95% CI: 0.47–1.04, *p* = 0.076; Figure 2D).

### 3.3. RELN Mutations in Predicting ICI Treatment Efficacy in NSCLC

**Figure 3:**
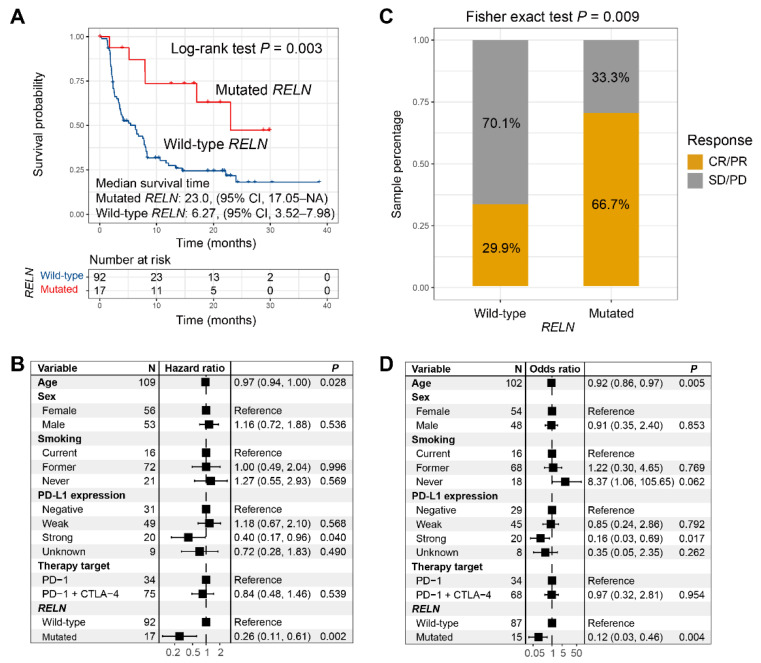
ICI treatment prognosis and response rate analyses of *RELN* mutations in NSCLC. (**A**) Survival curves of *RELN*-mutated and wild-type NSCLC patients. (**B**) Multivariable Cox regression analysis of *RELN* mutations was performed with clinical confounders taken into consideration. (**C**) Bar plot representation of ICI response rates of *RELN*-mutated and wild-type patients. (**D**) Multivariable logistic regression analysis of *RELN* mutations was achieved with multiple confounding variables adjusted.

A total of 36 (33.0%) of the 109 included NSCLC patients exhibited the ICI complete response or partial response statuses. *RELN* mutated in 17 (15.6%) of the above NSCLC patients. Survival analysis revealed that a significantly improved ICI survival benefit was found in NSCLC patients with *RELN* mutations (median survival time: 23.0 vs. 6.27 months, Log-rank test *p* = 0.003; Figure 3A). We incorporated multiple clinical confounding factors into a multivariable Cox regression analysis, and the association of *RELN* mutations with preferable ICI prognosis was still observed (HR: 0.26, 95% CI: 0.11–0.61, *p* = 0.002; Figure 3B). *RELN* mutation associations with ICI prognosis in diverse NSCLC ICI types are shown in Figure S5. Subsequent exploration indicated that an enhanced immunotherapeutic response rate was also found in *RELN*-mutated NSCLC patients (66.7% vs. 29.9%, Fisher exact test *p* = 0.009; Figure 3C). Multivariable adjusted analysis still confirmed this connection between *RELN* mutations and elevated ICI response rate (OR: 0.12, 95% CI: 0.03–0.46, *p* = 0.004; Figure 3D).

### 3.4. RELN Mutation Association with TMB

**Figure 4:**
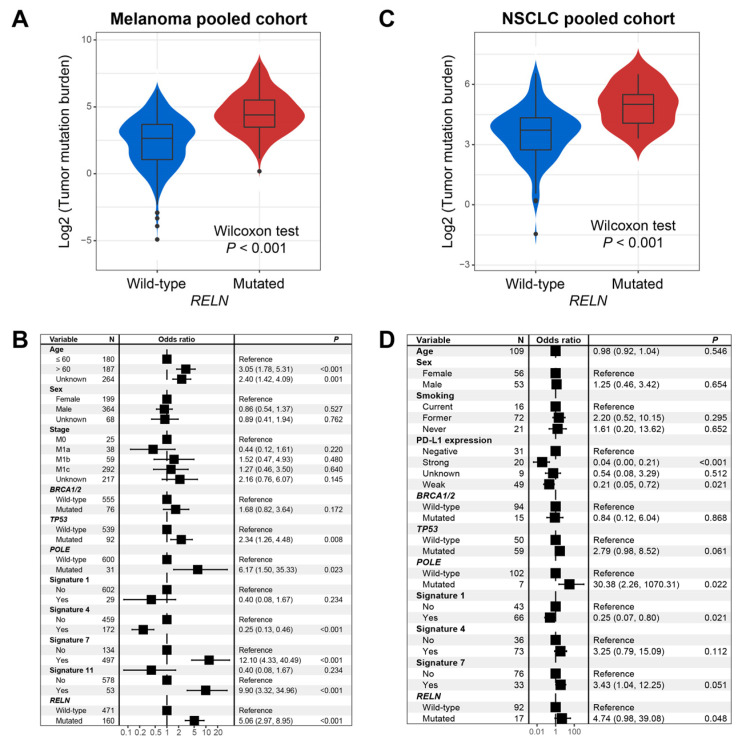
Association of *RELN* mutations with TMB in melanoma and NSCLC. (**A**) Univariate analysis between *RELN* mutations and TMB in melanoma. (**B**) Multivariable logistic analysis of *RELN* mutations was achieved with multiple confounding factors adjusted. (**C**) Univariate analysis between *RELN* mutations and TMB in NSCLC. (**D**) Multivariable logistic analysis of *RELN* mutations was achieved with multiple confounding factors controlled to acquire a real association.

In melanoma, genomic mutational analysis showed that patients with *RELN* mutations had a markedly higher TMB than *RELN* wild-type patients (Wilcoxon rank-sum test, *p* < 0.001; Figure 4A). Several recent studies have demonstrated that mutational signatures are linked with genomic instability and mutation rate. We therefore extracted four mutational signatures from melanoma mutation profiles; these were signatures 1, 4, 7, and 11 (Table S3). Subsequently, in order to adjust confounding factors and obtain a real association between *RELN* mutations and TMB, we conducted a multivariable logistic regression model with clinical variables, alterations in DNA repair genes, and four mutational signatures taken into account. The association of *RELN* mutations with elevated TMB was still significant (OR: 5.06, 95% CI: 2.97–8.95, *p* < 0.001; Figure 4B).

In NSCLC, we validated the association between *RELN* mutations and higher TMB (Wilcoxon rank-sum test, *p* < 0.001; Figure 4C). We also extracted three mutational signatures (i.e., signatures 1, 4, and 7) from NSCLC mutational profiles (Table S4). Consistently, a multivariable logistic regression model with confounders still confirmed that *RELN* mutations were linked with a significantly enhanced TMB (OR: 4.74, 95% CI: 0.98–39.08, *p* = 0.048; Figure 4D).

### 3.5. Immune Infiltration and Signaling Pathways Associated with RELN Mutations

**Figure 5:**
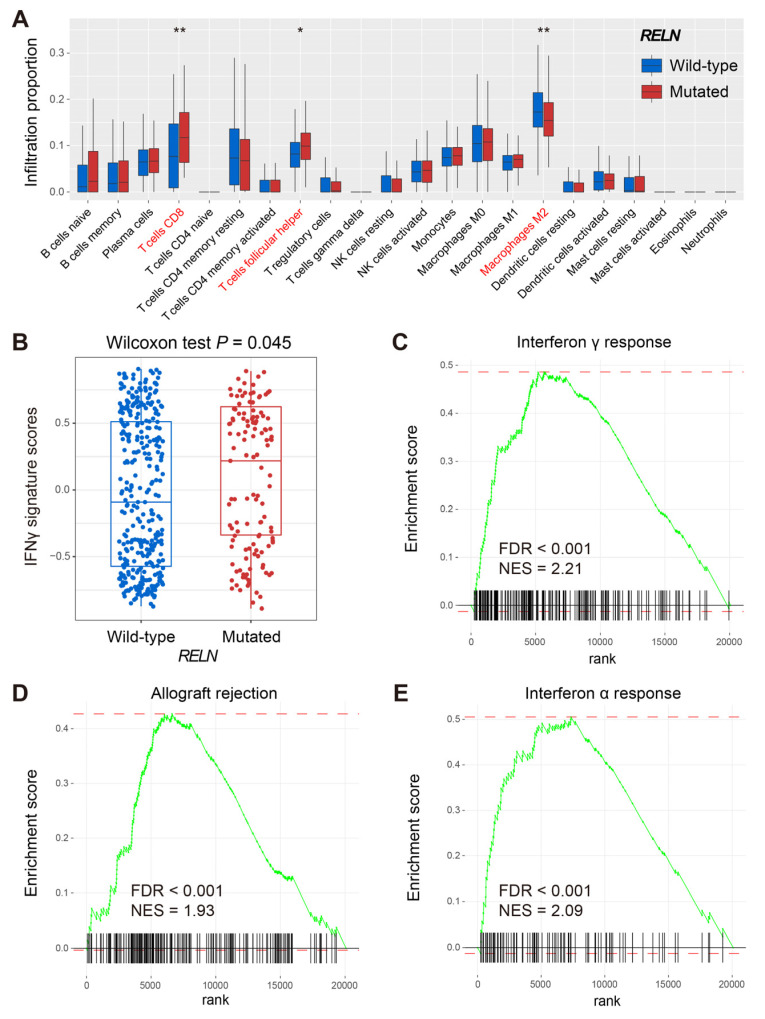
Immune infiltration and signaling pathways behind *RELN* mutations in melanoma. (**A**) CIBERSORT method revealed the distinct immunocyte infiltration in *RELN* two subgroups. (**B**) Distinct enrichment scores of IFNγ signature in *RELN* two subgroups. Immunogenicity-related signaling pathways of (**C**) interferon γ response, (**D**) allograft rejection, and (**E**) interferon α response were enriched in patients with *RELN* mutations. * *p* < 0.05, ** *p* < 0.01.

The CIBERSORT algorithm was used to evaluate distinct immunocyte infiltration levels between *RELN* subgroups in melanoma (Figure 5A). Results showed that pro-inflammatory immunocytes (e.g., CD8 T cells and T follicular helper cells) were significantly enriched in *RELN*-mutated melanoma patients (Wilcoxon rank-sum test, both *p* < 0.05). However, the infiltration abundance of immune-suppressive cells (e.g., M2 macrophages) was decreased in the subgroup mutated in this way (Wilcoxon rank-sum test, *p* < 0.01). The subsequent ssGSEA method revealed that patients with *RELN* mutations harbored significantly higher enrichment scores of IFNγ signature when compared with wild-type patients (Wilcoxon rank-sum test, *p* = 0.045; Figure 5B). GSEA results indicated that immunogenicity-related signaling pathways of interferon γ/α response and allograft rejection were observed in *RELN*-mutated melanoma patients (all FDR < 0.001; Figure 5C–E).

We also calculated the immunocyte infiltration level differences between two *RELN* subgroups in NSCLC. Consistently, favorable immunocyte infiltration was observed in patients with *RELN* mutations (Figure S6).

## 4. Discussion

Immunotherapies are clinically confirmed as promising cancer treatment strategies, especially for advanced or metastatic cancers. Although remarkable clinical benefits are observed, only a subset of patients is responsive. Therefore, newly identified biomarkers for evaluating ICI efficacy are needed immediately. In this work, we uncovered that *RELN* mutations were predictive of a better ICI treatment outcome and response in melanoma and NSCLC. Moreover, an elevated TMB and a favorable immune infiltration were also observed in patients with *RELN* mutations in both tumors. The above findings suggest that *RELN* mutations may be regarded as a possible indicator for assessing immunotherapeutic efficacy and used for selecting cancer patients to receive immune checkpoint-based therapies.

In our analysis, *RELN* mutations were found to be connected with the preferable ICI therapy outcome and response in both melanoma and NSCLC patients, which suggests the immunotherapeutic significance of *RELN* mutations in clinical practice. To explore whether *RELN* mutations play a role in other therapeutic types, we acquired mutational profiles and clinical features data of melanoma and NSCLC samples from the TCGA. Survival analysis demonstrated that no significant survival differences were noticed between *RELN*-mutated and wild-type subgroups in both tumors (Log-rank test *p* = 0.852 and 0.136, respectively; Figure S7). The above findings indicate that *RELN* mutations may play an efficacy predictive role in immunotherapeutic settings, rather than a prognostic role. Further analyses are necessary to elucidate the roles of *RELN* mutations in other treatment types.

Tumor mutation burden (TMB) has recently been reported as a promising molecular biomarker for evaluating ICI treatment outcome and response in several cancers [7,47,48,49]. Its high level is always correlated with favorable clinical ICI benefits. Nevertheless, the determination of TMB requires the performance of whole-exome sequencing, which is costly. On the other hand, the cut-off values for stratifying high and low TMB in diverse cancer types are distinct [50]. Several recent studies have revealed that mutations in a single gene, such as *POLE* [51], *TP53* [52], and *FAT1* [5], may be the potential surrogates for TMB. In this analysis, we observed that *RELN* mutations were related to an elevated TMB in both tumors. Taking into account that some confounding factors may influence the real association, we conducted multivariable-adjusted analyses to verify the association of *RELN* mutations with high TMB. The above findings suggest that *RELN* mutations may be regarded as a surrogate for TMB to evaluate immune treatment efficacy.

A favorable tumor microenvironment is important for the immune response and treatment efficacy [53]. Tumor-infiltrating immunocytes are vital elements in the microenvironment for regulating a series of biological processes [54]. We therefore explored the distinct immunocyte infiltration levels and signaling pathway distributions in *RELN*-mutated and wild-type groups. We observed that the higher infiltration abundance of CD8 T cells and the lower abundance of immune-suppressive M2 macrophages were enriched in melanoma patients with *RELN* mutations. Moreover, the immunogenicity-relevant pathways were also noticed in the group thus mutated. Consistently, in NSCLC, a preferable immunocyte infiltration and immune microenvironment were found in patients with *RELN* mutations. The above evidence showed that *RELN* mutations are predictive of better immune infiltration, which further supports the observed relationship between *RELN* mutation and favorable ICI treatment efficacy.

TMB and neoantigen burden have emerged as promising indicators for assessing ICI efficacy, and previous evidence has demonstrated their positive connection with the immunotherapy response rate and outcome via multiple clinical trials [15,47,55]. Nevertheless, a few studies concluded controversial results; that is, high TMB could not always accurately predict ICI response [47]. Immune checkpoints, such as PD-L1 expression, are another widely used biomarker linked with ICI therapies’ efficacy. Similarly, it may not work in some trials [56]. In view of the current situation, novel and more effective indicators are needed to distinguish subpopulations that are likely to be sensitive to ICI treatment.

A recent study has reported that *FAT1* mutations were associated with favorable ICI treatment efficacy in melanoma and NSCLC patients. To elucidate, *RELN* and *FAT1* mutations were two independent biomarkers for evaluating immunotherapeutic efficacy. We performed multivariable Cox regression models in melanoma and NSCLC cohorts with multiple confounding factors, including *RELN* and *FAT1* mutations, taken into account. We observed that both mutations exhibited preferable ICI treatment prognoses in melanoma and NSCLC patients after mutually adjusting (all HR < 1, all *p* < 0.05; Figures S8 and S9), which suggests that *RELN* and *FAT1* mutations are two independent biomarkers for predicting ICI response.

Some shortcomings exist in this study. First, the melanoma and NSCLC samples used in this study were acquired from publicly available databases and lacked in-house result validation. Second, the integrated immunogenomic cohorts were obtained based on several single cohorts, thus some biases may be introduced during data processing. Third, analyses on the transcriptomic level were performed by using gene expression data from TCGA cohorts, with no corresponding expression data for integrated cohorts.

## 5. Conclusions

Collectively, by leveraging genomic profiles and clinical information, *RELN* mutations were determined as a potential biomarker for ICI treatment efficacy prediction, which may provide some clues for selecting cancer patients to receive immunotherapies.

## Data Availability

All samples included in this work were publicly obtained and can be acquired by contacting the corresponding author under reasonable requests.

## Supplementary Materials

The following supporting information can be downloaded at: https://www.mdpi.com/article/10.3390/cells11233841/s1, Figure S1: mutational patterns of *RELN* and common melanoma driver genes exhibited with waterfall plot; Figure S2: detailed amino acid changes induced by *RELN* mutations in the integrated melanoma cohort; Figure S3: Kaplan-Meier survival analyses of *RELN* mutations in individual ICI-treated melanoma cohorts; Figure S4: Kaplan-Meier survival analyses of *RELN* mutations in distinct ICI treatment types in melanoma; Figure S5: Kaplan-Meier survival analyses of *RELN* mutations in individual ICI-treated NSCLC cohorts; Figure S6: distinct infiltration of 22 immunocytes of *RELN*-mutated and wild-type groups evaluated with CIBERSORT algorithm in NSCLC. Immunocytes highlighted with red are significantly differentially infiltrated; Figure S7: prognostic capacities of *RELN* mutations in (A) melanoma and (B) NSCLC patients derived from the TCGA project; Figure S8: Multivariable Cox regression analysis of RELN mutations was performedwith multiple clinical confounding factors taken into consideration in melanoma; Figure S9: Multivariable Cox regression analysis of RELN mutations was performed with multiple clinical confounding factors taken into consideration in NSCLC; Table S1: detailed clinical data and immunotherapy response information for 631 pooled melanoma patients; Table S2: detailed clinical data and immunotherapy response information for 109 pooled NSCLC patients; Table S3: the detected 4 mutational signatures with detailed mutational activities in the pooled melanoma cohort; Table S4: the detected 3 mutational signatures with detailed mutational activities in the pooled NSCLC cohort.
