# Supplementary material for: Identification and Validation of RELN Mutation as a Response Indicator for Immune Checkpoint Inhibitor Therapy in Melanoma and Non-Small Cell Lung Cancer
Source: Cells. 2022 Nov 30;11(23):3841. doi: 10.3390/cells11233841 (PMC9741468; doi:10.3390/cells11233841)
Supplement: Supplementary file 1 [file cells-11-03841-s001.zip › cells-1998833-supplementary/Supplementary Figures.pdf]

# Supplementary Figures

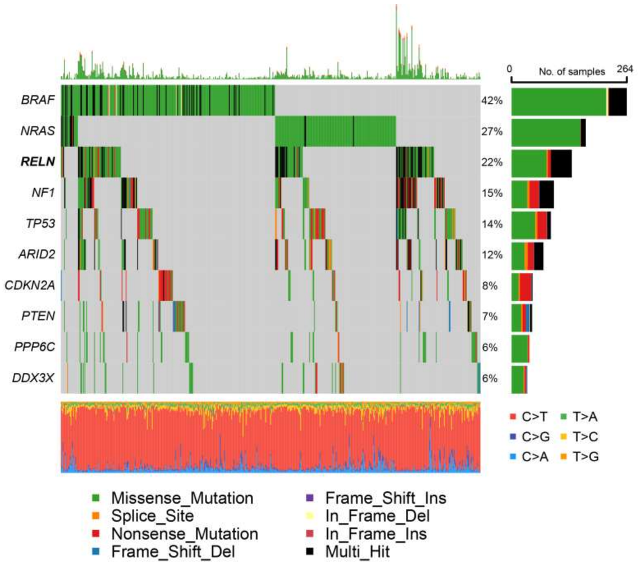


**Figure S1.** Mutational patterns of *RELN* and common melanoma driver genes exhibited with waterfall plot.

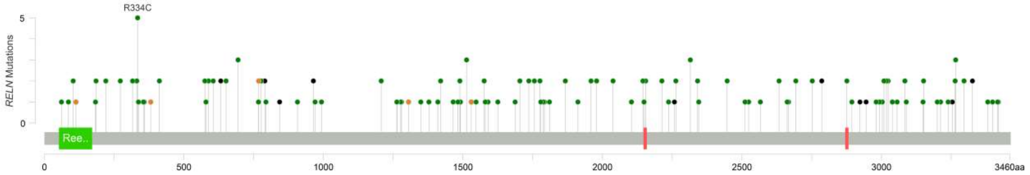


**Figure S2.** Detailed amino acid changes induced by *RELN* mutations in the integrated melanoma cohort.

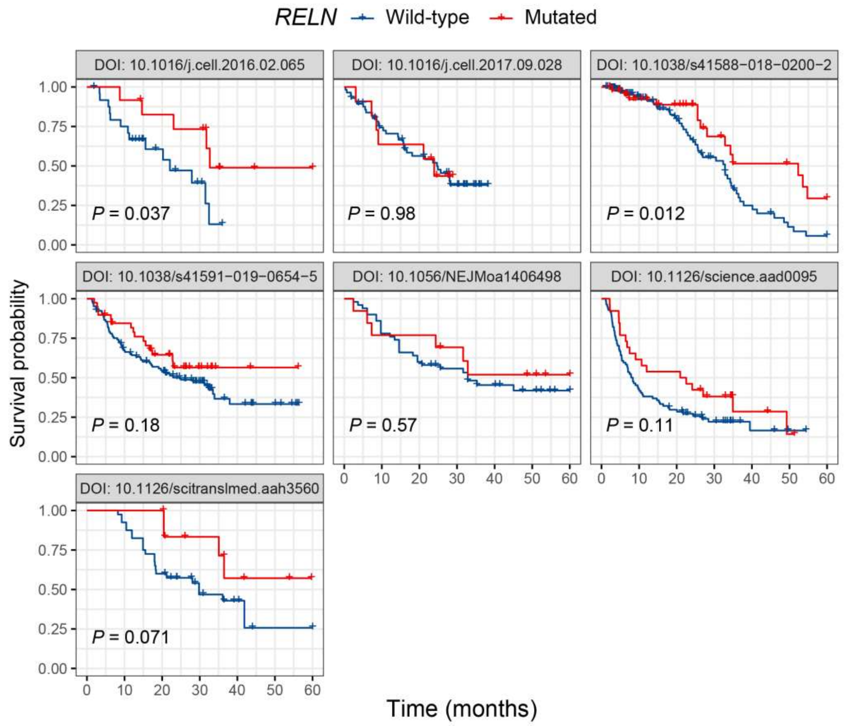


**Figure S3.** Kaplan-Meier survival analyses of *RELN* mutations in individual ICI-treated melanoma cohorts.

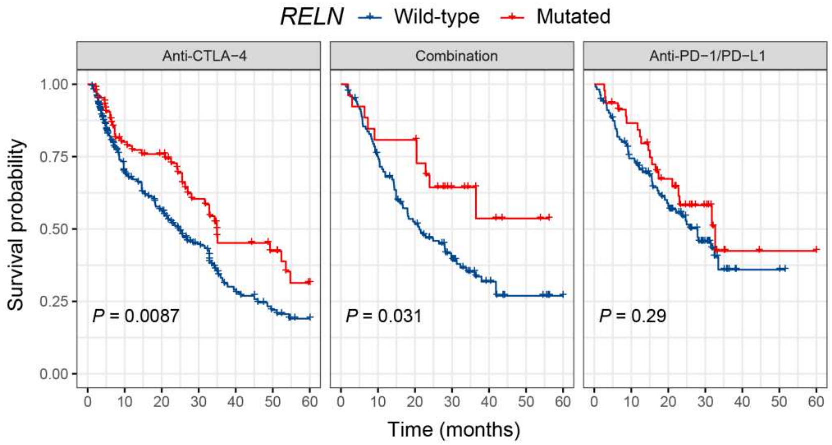


**Figure S4.** Kaplan-Meier survival analyses of *RELN* mutations in distinct ICI treatment types in melanoma.

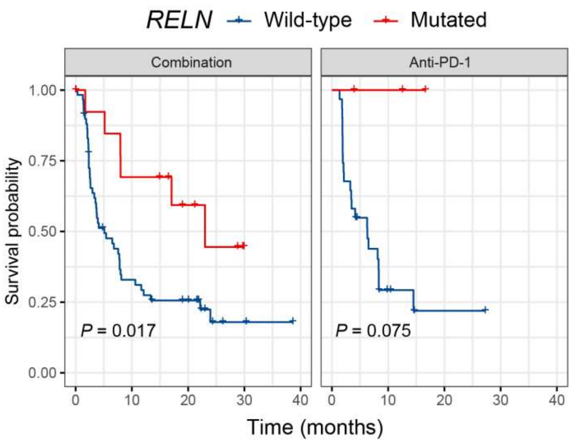


**Figure S5.** Kaplan-Meier survival analyses of *RELN* mutations in individual ICI-treated NSCLC cohorts.

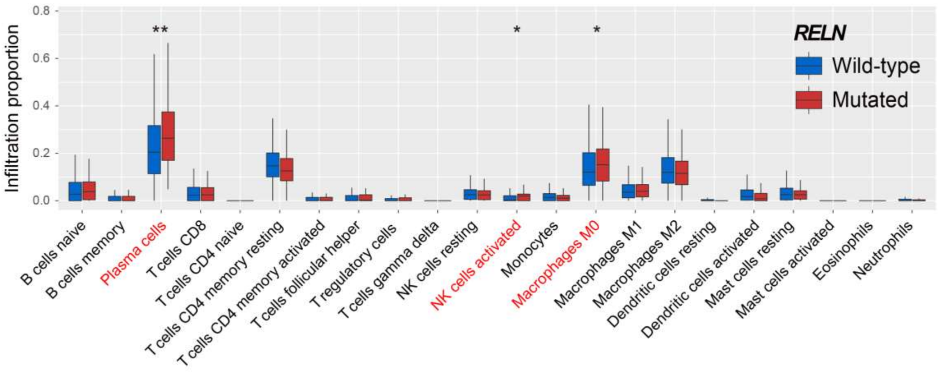


**Figure S6.** Distinct infiltration of 22 immunocytes of *RELN* mutated and wild-type groups evaluated with CIBERSORT algorithm in NSCLC. Immunocytes highlighted with red are significantly differentially infiltrated.

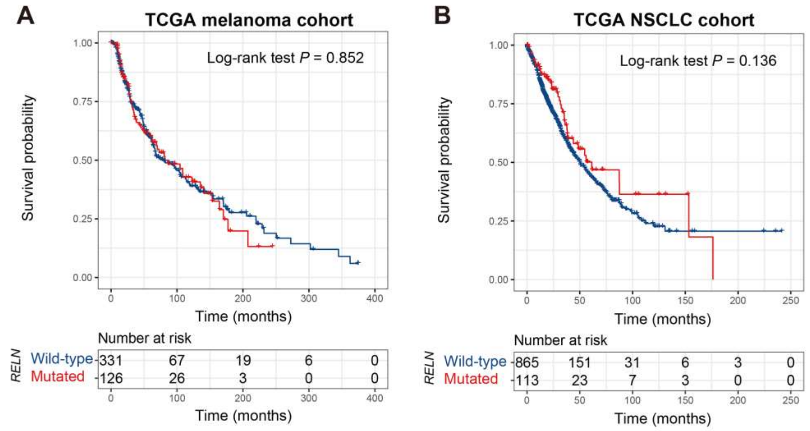


**Figure S7.** Prognostic capacities of *RELN* mutations in (A) melanoma and (B) NSCLC patients derived from the TCGA project.

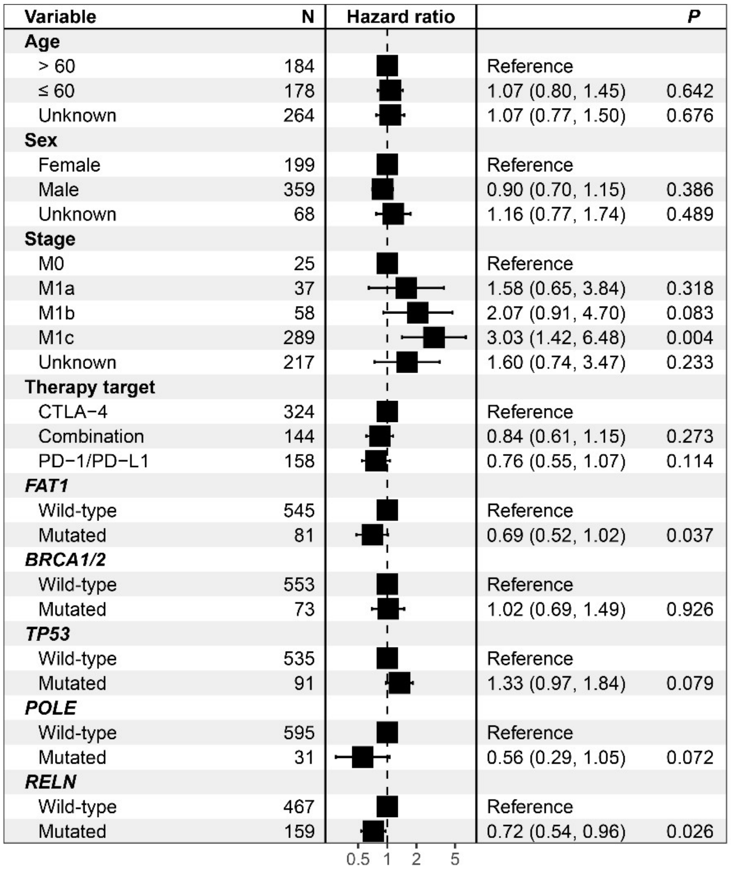


**Figure S8.** Multivariable Cox regression analysis of *RELN* mutations was performed with multiple clinical confounding factors taken into consideration in melanoma.

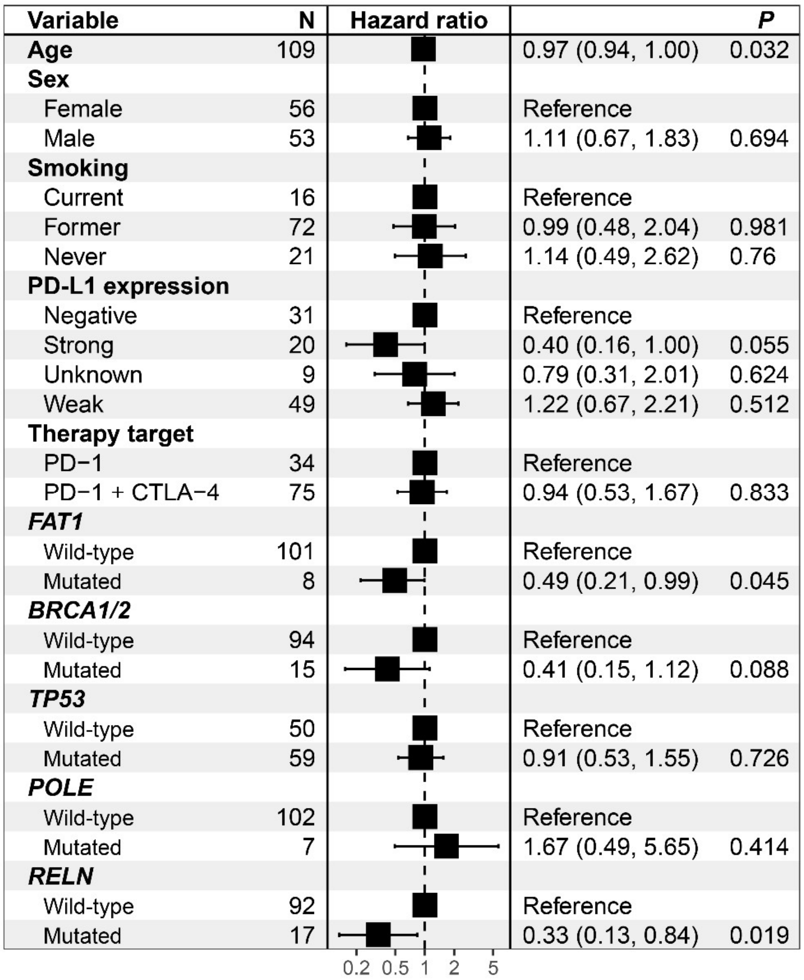


| Variable | N | | *P* |
|---|---|---|---|
| **Age** | 109 | 0.97 (0.94, 1.00) | 0.032 |
| **Sex** | | | |
| Female | 56 | Reference | |
| Male | 53 | 1.11 (0.67, 1.83) | 0.694 |
| **Smoking** | | | |
| Current | 16 | Reference | |
| Former | 72 | 0.99 (0.48, 2.04) | 0.981 |
| Never | 21 | 1.14 (0.49, 2.62) | 0.76 |
| **PD-L1 expression** | | | |
| Negative | 31 | Reference | |
| Strong | 20 | 0.40 (0.16, 1.00) | 0.055 |
| Unknown | 9 | 0.79 (0.31, 2.01) | 0.624 |
| Weak | 49 | 1.22 (0.67, 2.21) | 0.512 |
| **Therapy target** | | | |
| PD−1 | 34 | Reference | |
| PD−1 + CTLA−4 | 75 | 0.94 (0.53, 1.67) | 0.833 |
| ***FAT1*** | | | |
| Wild-type | 101 | Reference | |
| Mutated | 8 | 0.49 (0.21, 0.99) | 0.045 |
| ***BRCA1/2*** | | | |
| Wild-type | 94 | Reference | |
| Mutated | 15 | 0.41 (0.15, 1.12) | 0.088 |
| ***TP53*** | | | |
| Wild-type | 50 | Reference | |
| Mutated | 59 | 0.91 (0.53, 1.55) | 0.726 |
| ***POLE*** | | | |
| Wild-type | 102 | Reference | |
| Mutated | 7 | 1.67 (0.49, 5.65) | 0.414 |
| ***RELN*** | | | |
| Wild-type | 92 | Reference | |
| Mutated | 17 | 0.33 (0.13, 0.84) | 0.019 |

**Figure S9.** Multivariable Cox regression analysis of *RELN* mutations was performed with multiple clinical confounding factors taken into consideration in NSCLC.
